# Model Parameters and Outbreak Control for SARS

**DOI:** 10.3201/eid1007.030647

**Published:** 2004-07

**Authors:** Gerardo Chowell, Carlos Castillo-Chavez, Paul W. Fenimore, Christopher M. Kribs-Zaleta, Leon Arriola, James M. Hyman

**Affiliations:** * Los Alamos National Laboratory, Los Alamos, New Mexico, USA;; † Cornell University, Ithaca, New York, USA;; ‡ Arizona State University, Tempe, Arizona, USA;; § University of Texas at Arlington, Arlington, Texas, USA

**Keywords:** SARS, uncertainty, sensitivity, diagnosis, isolation, reproductive number, research

## Abstract

Control of the 2002–2003 severe acute respiratory syndrome (SARS) outbreak was based on rapid diagnosis coupled with effective patient isolation. We used uncertainty and sensitivity analysis of the basic reproductive number *R_0_* to assess the role that model parameters play in outbreak control. The transmission rate and isolation effectiveness have the largest fractional effect on *R_0_*. We estimated the distribution of the reproductive number *R_0_* under perfect isolation conditions. The distribution lies in the interquartile range 0.19–1.08, with a median of 0.49. Even though the median of *R_0_* is <1, we found that 25% of our *R_0_* distribution lies at *R_0_* > 1, even with perfect isolation. This implies the need to simultaneously apply more than one method of control.

## Abstract 2

Tool for estimating basic reproductive number for the SARS outbreak suggests need for multiple methods of control.

Severe acute respiratory syndrome (SARS), a viral respiratory disease, has been reported in 32 countries as of July 11, 2003. SARS is believed to have originated in Guangdong Province, China, in November 2002 (1). Researchers at the Erasmus Medical Center in Rotterdam, the Netherlands, identified a coronavirus as the agent responsible for infecting 8,437 persons worldwide, with 813 deaths as of July 11, 2003 (2). According to recent epidemiologic data from Hong Kong (3), a person exposed to SARS enters an incubation period with a mean length of 6.4 days. Symptomatic persons in that study were hospitalized at a mean rate of 1/4.85 days^–1^. Those who recovered were discharged a mean of 23.5 days after diagnosis, while the mean period to death was 35.9 days after diagnosis. Because no specific treatment for SARS exists, control of the epidemic relied on rapid diagnosis and isolation of patients (1), an approach that is reported to be effective (4). However, most early SARS cases in Toronto occurred in hospitals, with movement of SARS patients between hospitals contributing to the disease's initial spread (5). In Taiwan, 94% of SARS cases occurred through transmission in hospital wards (6), and similar effects occurred in Hong Kong and Singapore (7). Although the SARS epidemic was eventually controlled, the measures used to achieve that control varied greatly in scope from one place to another. Control of an outbreak relies partly on identifying what disease parameters are likely to lead to a reduction in the reproduction number *R_0_*. Here we calculate the dependence of *R_0_* on model parameters.

## Methods

Two models of the SARS epidemic that incorporate the effects of quarantine and early detection of new cases but assume perfect isolation were recently introduced (8,9). A slightly different model was used to quantify the role that fast diagnosis and efficient isolation of patients played in Toronto's outbreak (10). This model predicted control in Toronto and showed that lack of immediate action would have been catastrophic (11). The model incorporates differences in the population's susceptibility (3) by dividing the population into classes S_1_ (high risk) and S_2_ (low risk). A low-risk group in the age range <19 years can be observed from the age-specific incidence in Hong Kong (3). The low-risk class (S_2_) has a reduced susceptibility to SARS, measured by the parameter p (0 < p < 1). While p = 0 denotes no susceptibility to SARS, p = 1 indicates that both susceptible classes are equally susceptible to SARS. Initially, S_1_ = rN and S_2_ = (1-ρ)N, where N is the total population size and r is the initial proportion of fully susceptible (S_1_) persons. Susceptible persons exposed to SARS enter the exposed class (assumed to be asymptomatic) with a rate proportional to β and remain there for a mean incubation period of 1/k. The possibility of reduced transmission from the exposed class is included through the parameter q (0 < q < 1), a relative measure of infectiousness. Once symptomatic, exposed persons progress to the infectious class (illness not yet diagnosed), where they may recover at the rate γ_1_, die at rate δ, or enter the diagnosed class at rate α. Isolation mechanisms may be put in place in the diagnosed class to reduce their impact on transmission. The relative infectiousness after isolation has begun is measured by the parameter *l* (0 < *l* < 1) so that *l* = 0 denotes perfect isolation and *l* = 1 denotes ineffective isolation.

### Basic Reproductive Number (*R_0_*)

The basic reproductive number (*R_0_*) is the average number of secondary cases generated by a primary case. If *R_0_* < 1, an epidemic can not be sustained. On the other hand, if *R_0_* > 1, an epidemic typically occurs.

The basic reproductive number derived from our model (10) is given by the formula

_
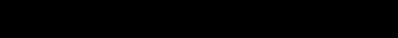
_.

This equation includes 10 parameters of which 2, the diagnostic rate (α) and the relative infectiousness during isolation (*l*), are widely recognized as being amenable to modification by medical intervention. The transmission rate (β) is defined as the number of persons infected per infectious person per day. This differs from *R_0_*, which is the average number of secondary cases that an infectious person generates when introduced into a susceptible population. Definitions for the remaining parameters are provided in Table 1.

**Table 1:** An extended definition for the transmission rate (β) is the number of persons infected per infectious person per day while the basic reproductive number (*R_0_*) is the average number of secondary cases an infectious individual can generate when this rate is introduced into a susceptible population

Parameter	Definition	Baseline value
p^a^	Reduction in risk of infection for class S_2_	0.33
ρ^a^	Initial proportion of the population at higher risk for SARS	0.77
β^b^	Transmission rate per day	0.25
1/k^a^	Mean incubation period (days)	6.37
1/γ_1_	Mean infectious period (days)	28.4
1/γ_2_^a^	Mean infectious period for persons with diagnosed SARS (days)	23.5
1/α	Mean period before diagnosis (days)	4.85
δ^a^	Induced death rate per day	0.0279
q	Relative measure of infectiousness for the exposed class	0.1
*l* ^c^	Relative infectiousness after isolation has begun	[0,1]

^a^Baseline values for k, γ_2_, α, ρ, p and δ have been taken from reference 3. ^b^β = 0.25 is our estimated transmission rate in Hong Kong. ^c^*l* = 0 means perfect isolation, while *l* = 1 means no isolation.

### Parameter Estimation

Baseline values for k, γ_2_, δ, and α are taken from the mean values estimated in reference 3. Because whether asymptomatic persons (exposed class) can transmit the disease is not known, we have fixed q = 0.1 (the relative infectiousness of exposed, asymptomatic persons) as in reference 10.

The model parameters Θ = (β, *l*) are fitted to Hong Kong data (2) by least squares fit to the cumulative number of cases C (t, Θ) (equation 1 in reference 10). All other parameters are fixed to their baseline values (Table 1). We used a computer program (Berkeley Madonna, R.I. Macey and G.F. Foster, Berkeley, CA) and appropriate initial conditions for the parameters for the optimization process, which was repeated 10 times (each time the program is fed with two different initial conditions for each parameter) before the "best fit" was chosen. The best fit gives β = 0.25 and *l* = 0.43. We also estimated the relative infectiousness after isolation (*l*) for the case of Singapore (*l* = 0.49) by following the least squares procedure described above. However, for the case of Toronto, not enough data were available on the initial growth of the outbreak. Hence, we only estimated *l* from Toronto data after control measures were put in place on March 26 (10,11), where *l* = 0.1. We used the transmission rate (β) obtained from Hong Kong data as the baseline value (Table 1).

We revised earlier estimates for ρ and p (10) (both affect *R_0_*) using data from the age distribution of residents and the age-specific incidence of SARS in Hong Kong, as reported (3). The revised estimates are ρ = 0.77 (the initial proportion of the population at higher risk) and p = 1/3 (the measure of reduced susceptibility in S_2_). The lower-risk subpopulation lies in the age range <19. It constitutes approximately 23% of Hong Kong's population (3). The fact that most of the SARS cases included in the epidemiologic studies of the Toronto outbreak (5) were transmitted in hospitals limits the use of general demographic data from Toronto in the estimation of ρ and p. Hence, we used the parameters estimated from the situation in Hong Kong. Baseline values for all the parameters are given in Table 1.

### Uncertainty Analysis for *R_0_*

**Figure 1:**
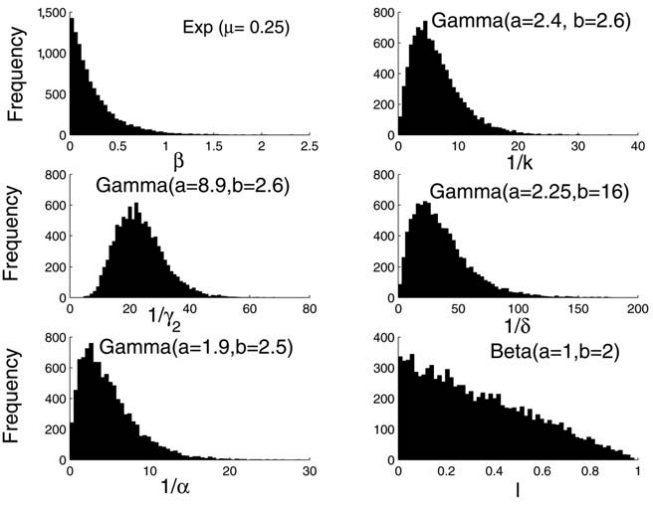
Histograms of the six distributed parameters appearing in equation 1 with sample size 10^5^. The transmission rate was assumed to be exponentially distributed with mean 0.25, our estimated transmission rate in Hong Kong. Here *l* is assumed to have a beta distribution (*l* ~ β [1,2]). Alternative distributions for *l* were also used as described in the text. All other distributions were taken from reference 3.

We carried out an uncertainty analysis on the basic reproductive number (*R_0_*) to assess the variability in *R_0_* that results from the uncertainty in the model parameters. We used a Monte Carlo procedure (simple random sampling) to quantify the uncertainty of *R_0_* to model parameters when these parameters are distributed. Similar methods have been used before (12–14). Parameters (k, γ_2_, δ, α) were assigned a different probability density function (PDF) (Figure 1), which is taken from reference 3. The relative measure of infectiousness of persons after isolation procedures are put in place (*l*) was assumed to be uniformly distributed in the interval (0 < *l* < 1). The observed heterogeneity in transmission rates during the SARS epidemic is modeled here by assuming that β is distributed exponentially with mean 0.25 person^–1^ day^–1^ (our estimate of the transmission rate in Hong Kong). Parameters q, p, and ρ are fixed to their baseline values (Table 1). We sampled the set of six parameters (β, k, γ_2_, δ, α, *l*) 10^5^ times, holding q, p, and ρ fixed. We then computed *R_0_* from each set. A probability density function for *R_0_* is obtained and can be statistically characterized. Here, we characterize *R_0_* by its median and interquartile range.

### Sensitivity Analysis for *R_0_*

We performed a sensitivity analysis on *R_0_* to quantify the effect of changes in the model parameters on *R_0_*. Hence, we rank model parameters according to the size of their effect on *R_0_*. Partial rank correlation coefficients (12–15) were computed between each of the parameters (with the exception of p, q, and ρ, which were held fixed) and *R_0_* as samples were drawn from the distributions, thus quantifying the strength of the parameter's linear association with *R_0_*. The larger the partial rank correlation coefficient, the larger the influence of the input parameter on the magnitude of *R_0_*. Because the distribution of the parameter *l* (relative infectiousness after isolation) is not known, we also studied the sensitivity of *R_0_* to various distributions of *l*. Distributions of *l* used for the Monte Carlo calculation of the partial rank correlation coefficients are: a) *l* ~ β (a = 2, b = 2) where β is used to denote a beta distribution. Here, the likelihood of *l* is bell-shaped with mean 0.5 and variance 0.05; b) *l* ~ β (a = 1, b = 2), the likelihood of *l* decreases linearly in the [0,1] interval; and c) *l* ~ β (a = 2, b = 1), the likelihood of *l* increases linearly in the [0,1] interval.

## Results

### Uncertainty Analysis for *R_0_*

**Figure 2:**
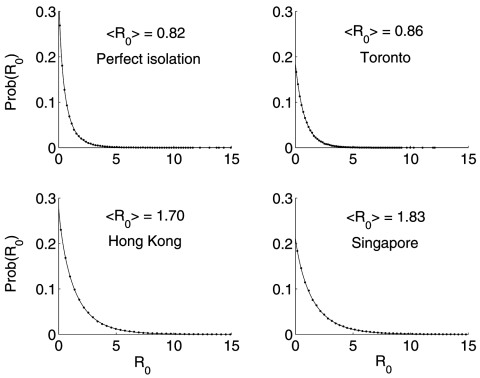
Empiric (dots) and stretched exponential estimated probability density function Prob(*R_0_*) = a exp[–(*R_0_*/b)^c^] (solid line) (16) of *R_0_* for the cases of Toronto (a = 0.186, b = 0.803, c = 0.957, after control measures had been implemented), Hong Kong (a = 0.281, b = 1.312, c = 0.858), and Singapore (a = 0.213, b = 1.466, c = 0.883) obtained from our uncertainty analysis. The distribution for the case of perfect isolation (*l* = 0, a = 0.369, b = 0.473, c = 0.756) is shown as a reference.

**Figure 3:**
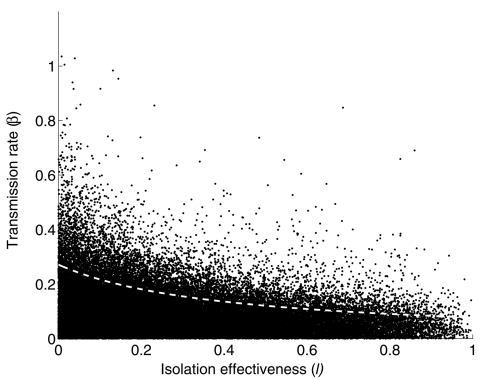
(β, *l*) parameter space when *R_0_* < 1 obtained from the uncertainty analysis (black dots). The deterministic (β, *l*) level curve when *R_0_* = 1 is shown in by the dotted white line. All other parameters in equation 1 were fixed to their baseline values (Table 1). *l* = 0 denotes perfect isolation; *l* = 1 denotes no isolation.

The resulting *R_0_* distribution lies in the interquartile range 0.43–2.41, with a median of 1.10. Moreover, the probability that *R_0_* > 1 is 0.53. The same Monte Carlo procedure, but with fixed values of *l* = 0.1 and α = 1/3 day^–1^ for Toronto (i.e., after implementing control measures on March 26), give a median and interquartile range for the distribution of *R_0_* = 0.58 (0.24–1.18) (Table 2). Similarly, a lower rate of diagnosis a = 1/4.85 day^–1^ and the relative infectiousness after isolation in Hong Kong (*l* = 0.43) and Singapore (*l* = 0.49) gives *R_0_* = 1.10 (0.44–2.29) and 1.17 (0.47–2.47), respectively (Figure 2). Perfect isolation (*l* = 0) gives *R_0_* = 0.49 (0.19–1.08). Especially noteworthy is that even in cases when eventual control of an outbreak is achieved (Toronto and a hypothetical case of perfect isolation), 25% of the weight of the distribution of *R_0_* lies at *R_0_* > 1. Furthermore, the median and interquartile range of *R_0_* are larger when p = 1, as has been assumed (8). In Figure 3 we show the (β, *l*) parameter space when *R_0_* < 1 obtained from our uncertainty analysis (14).

**Table 2:** The median and the interquartile range (IQR) of the distribution of the basic reproductive number (*R_0_*) of SARS for Toronto, Hong Kong, and Singapore obtained from our uncertainty analysis

Location	*R_0_* mean	*R_0_* median	*R_0_* IQR
Toronto, Canada (*l* = 0.10)	0.86	0.58	0.24–1.18
Hong Kong (*l* = 0.43)	1.70	1.10	0.44–2.29
Singapore (*l* = 0.49)	1.83	1.17	0.47–2.47

### Sensitivity Analysis for *R_0_*

**Figure 4:**
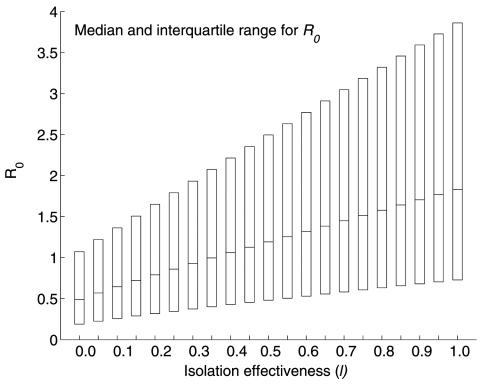
Boxplot of the sensitivity of *R_0_* estimates to varying values of *l*, the relative infectiousness after isolation has begun. *l* = 0 denotes perfect isolation while *l* = 1 denotes no isolation. The boxplot shows the median and the interquartile range of *R_0_* obtained from Monte Carlo sampling of size 10^5^.

The transmission rate β and the relative infectivity during isolation (*l*) are the most influential parameters in determining *R_0_*. The systematic decline in *R_0_* with increasing *l* in the range [0,1] is illustrated in Figure 4. Furthermore, our results do not change if we assume the three distributions mentioned in the Methods section (sensitivity analysis) for the parameter *l*. Table 3 shows the partial rank correlation coefficients for the other three possible distributions of *l*. The transmission rate is ranked first independent of the distribution of *l*. The relative infectiousness after isolation is ranked second when *l* comes from distributions (a) and (b) and ranked third when it comes from distribution (c) (see Methods). Our sensitivity analysis is corroborated by computing local derivatives on *R_0_* (see Appendix). Because bounds exist on how much a given parameter can change in practice, achieving control (i.e., *R_0_* < 1) can require changing parameters other than those with the highest partial rank correlation coefficient. For example, reference 10 showed that control of the outbreak in Toronto relied on both a reduction in *l* and 1/α, even though α is ranked fairly low by the partial rank correlation coefficient.

**Table 3:** Partial rank correlation coefficients (PRCCs) between each of the input parameters and *R_0_* from Monte Carlo sampling of size 10^5^ for different distributions of the relative infectiousness after isolation (*l*) as described in the text

Probability distribution	Input parameters in order of decreasing PRCC (shown in parenthesis)
β (a = 2, b = 2)	β (0.92), *l* (0.57), δ (0.53), γ_2_ (0.35), α (0.32), k (0.13)
β (a = 1, b = 2)	β (0.90), *l* (0.60), δ (0.44), α (0.39), γ_2_ (0.26), k (0.12)
β (a = 2, b = 1)	β (0.92), δ (0.60), *l* (0.51), γ_2_ (0.40), α (0.22), k (0.11)

## Conclusion

We have estimated *R_0_* for the cases of Toronto, Hong Kong, and Singapore (Table 2) through an uncertainty analysis shown in equation 1. Our estimates for *R_0_* agree with the empirical *R_0_* observed from the data of the first week of the SARS outbreak in Singapore (8). A stretched exponential distribution fits the resulting distributions of *R_0_* for the different locations (Figure 2). Even though the median of *R_0_* is <1 when perfect patient isolation is assumed (*l* = 0), we find that 25% of our *R_0_* distribution lies at *R_0_* > 1. That is, implementing a single method for control may not be sufficient to contain a SARS outbreak. Control may require modifying more than one parameter amenable to intervention. In our model, these parameters include the diagnostic rate (α), the relative infectiousness after isolation has begun (*l*), and the per capita transmission rate (β). The fact that α and *l* are not independent, but are tightly coupled, favors control.

Our expression for *R_0_* incorporates the effects of diagnosis-isolation strategies. Moreover, our approach includes differential susceptibility (p) and effective population size (ρ). Most models take p = 1, even though data from Hong Kong show that a low-risk subpopulation lies in the age range <19, approximately 23% of Hong Kong's population (3). The assumption p = 1 thus overestimates *R_0_*.

Our sensitivity analysis shows that the transmission rate (β) and the relative infectiousness after isolation in hospitals (*l*) have the largest effect on *R_0_*. With the exception of a few measures, such as closing schools, no clear policies would modify β directly. This means that a substantial effort must be (and has been) made by the medical community to modify other parameters, such as the diagnostic rate. Hence, the strong sensitivity of *R_0_* to the transmission rate β indicates that efforts in finding intervention strategies that manage to systematically lower the contact rate of persons of all age groups promise an effective means for lowering *R_0_*. Such strategies may include using face masks (the probability of transmission per contact may be reduced), washing hands, and avoiding large crowds (large public events).

Associated with the role of screening, diagnosis, and the effective isolation of patients is the issue of cost. We cannot ignore or minimize the value of stringent quarantine measures and the probability of compliance combined with the economic effect of lost wages (thousands were quarantined in Taiwan, Hong Kong, and Singapore [17]), the costs associated with screening at airports and hospitals, the cost associated with closing hospitals; and the costs associated with isolating SARS patients and exposed persons (see Appendix for a brief discussion).

## Appendix

### Local Sensitivity Analysis of the Basic Reproductive Number

The sensitivity analysis approach through an exhaustive sampling of the parameter space provides a global measure of the sensitivity of model parameters. Another approach is to compute the sensitivity indices of the model parameters through local derivatives (18). This approach only provides a local measure as the sensitivity indices can change when the parameter values change. Here, we use local sensitivity analysis to corroborate our global sensitivity analysis results and discuss how this approach can be applied in the analysis of cost as part of a policy of outbreak control.

Let λ represent any of the 10 nonnegative parameters, β, ρ, p, q, k, γ_1_, γ _2_, δ, α, and *l*, that define the basic reproductive number of our model (19)


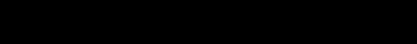

(1)

If a "small" perturbation δλ is made to the parameter λ, a corresponding change will occur in *R_0_* as δ*R_0_*, where


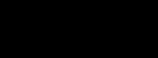



The normalized sensitivity index Ψ_λ_ is the ratio of the corresponding normalized changes and is defined as


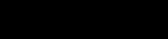

(2)

An approximation of the perturbed value of *R_0_*, in terms of the sensitivity index is


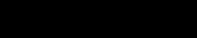



where the 10 normalized sensitivity indexes are


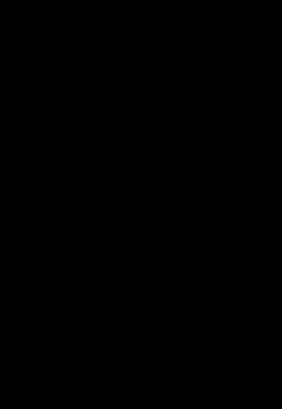



with η = p(1–ρ)+ρ and γ_2_ = a γ_1_/( α –γ_1_). For the values of the parameters used in this model, the sensitivity indices Ψ_β_, Ψ_ρ_, Ψ_p_, Ψ_q_, and Ψ*_l_* are positive, Ψ_k_ = –Ψ_q_, and the remaining indexes are negative. Furthermore, since all of the indexes (except Ψ_β_) are functions of the parameters, the sensitivity indexes will change as the parameter values change.

For our specific case where β = 0.25, q = 0.1, p = 1/3, k = 0.15707, α = 0.2061, γ_1_ = 0.035285, γ_2_ = 0.0426, δ = 0.0279, and ρ = 0.77 and Toronto (*l* = 0.1) or Hong Kong (*l* = 0.43), the normalized sensitivity indices are computed. The sensitivity indices and the associated percentage changes needed to affect a 1% decrease in *R_0_* are given in Tables A1 and A2. Since the effective rate of patient isolation and the average rate of diagnosis are two feasible intervention strategies, we examine how changes to the parameters *l* and α affect *R_0_*.

Let us first consider the outbreak in Hong Kong. The value α = 0.2061 means that the mean time to diagnose an infected person's illness is approximately 4.85 days. The sensitivity index Ψ_α_ = –0.1933 means that a 5.2% increase in α, which in turn requires a decrease of 5.7 hours of mean time to diagnosis, would result in a decrease of approximately 1% in *R_0_*. Similarly, the sensitivity index Ψ*_l_* = 0.5183 suggests that a 1.9% decrease in the value of *l*, that is going from 0.43 to 0.42 isolation effectiveness,^2^ results in a 1% decrease in *R_0_*. In other words, individually a 5.2% increase in a or a 1.9% decrease in *l* both result in approximately a 1% decrease in *R_0_*. For the particular values of the parameters chosen for Hong Kong, the most effective way to reduce *R_0_* is to decrease the transmission rate β and the parameter *l* (improve the effective isolation rate). In the case of Toronto, Ψ_α_ = –0.4758 means that a 2.1% increase in α, results in a 1% decrease in *R_0_*, whereas Ψ*_l_* = 0.2001 means a 5% decrease in *l* also results in a 1% decrease in *R_0_*.

As can be seen from these two examples, the importance or ranking of the sensitivity indices can change as the values of the parameters change. Specifically, the sensitivity indices Ψ*_l_* and Ψ_α_ satisfy the relationship


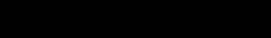

(3)

**Figure A1:**
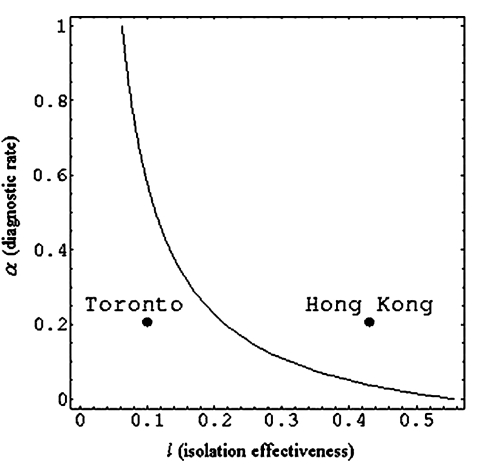
Level curve of (*l*,α), where *l*(α+2γ_1_+2δ) = δ+γ_2_.

For the particular values of the parameters given above, the Figure A1 shows the level curve for the pair (*l*,α), where *l*(α+2γ_1_+2δ) = δ+γ_2_. The particular parameter values are either for Toronto (*l,*α) = (0.1, 0.2064) or for Hong Kong (*l,*α) = (0.43, 0.2064). Choosing the parameter values (*l,*α) below the level curve means that ||Ψ*_l_*|| < ||Ψ_α_||and the converse is true if (*l,*α) is chosen above the curve. Along the level curve, the magnitude of the sensitivities is equal. Notice that the level curve divides the parameter space into two regions, each of area A_above_ and A_below_, respectively. Since A_above_ >> A_below_, Ψ*_l_* will be the dominant sensitivity index for randomly chosen (*l,*α).

One aspect of implementing an efficient intervention policy is the fact that limited resources are available. If one assumes, for example, that the strategies of isolation and diagnosis have associated 1% incremental costs in implementation of δC_I_ and δC_D_, respectively, then a mixed strategy should be formulated that maximizes the effectiveness of a combined intervention. Specifically, if *x* denotes the magnitude of percentage decrease in *l*, and *y* denotes the % increase in a and assuming that there is a maximum amount of total additional resources available (δC_T_), then the total additional cost of a new mixed isolation and diagnosis intervention policy must satisfy the inequality δC_I_*x*+δC_D_*y* < δC_T_. Since the objective is to maximize the decrease in *R_0_*, this means we want to maximize the objective function P = ||Ψ*_l_*||*x*+||Ψ_α_||*y* under the appropriate constraints. In a more general setting, additional nonlinear constraints could be involved, which case would require one to solve a nonlinear optimization problem. The situation in which the cost of diagnosis of infected persons may be much greater than the cost of isolation or vice versa is certainly of interest.

## Footnotes

2 Recall that *l* = 0 corresponds to complete isolation, whereas *l* = 1 means no effective isolation occurs. Hence, a decrease in *l* means an increase in the effective isolation of the infected persons.

## Appendix

**Table A1:** Sensitivity indices for Toronto with *l* = 0.1

Positive sensitivity indices		Negative sensitivity indices	
Ψ_β_ = 1	–1%	Ψ_α_ = –0.4758	2.10%
Ψ_r_ = 0.6063	–1.65%	Ψ_δ_ = –0.1707	5.86%
Ψ*_l_* = 0.2001	–4.99%	Ψ_γ2_ = –0.1208	8.28%
Ψ_γ_ = 0.1172	–8.53%	Ψ_k_ = –0.1172	8.53%
Ψ_p_ = 0.0906	–11.04%	Ψ_γ1_ = –0.1156	8.65%

**Table A2:** Sensitivity indices for Hong Kong with *l* = 0.43

Positive sensitivity indices		Negative sensitivity indices	
Ψ_β_ = 1	–1%	Ψ_γ2_ = –0.3129	3.19%
Ψ_ρ_ = 0.6063	–1.65%	Ψ_δ_ = –0.3016	3.32%
Ψ*_l_* = 0.5183	–1.93%	Ψ_α_ = –0.1933	5.17%
Ψ_p_ = 0.0906	–11.04%	Ψ_γ1_ = –0.1216	8.22%
Ψ_q_ = 0.0706	–14.16%	Ψ_k_ = –0.0706	14.16%
